# Mild cognitive impairment and deficits in instrumental activities of daily living: a systematic review

**DOI:** 10.1186/s13195-015-0099-0

**Published:** 2015-03-18

**Authors:** Katrin Jekel, Marinella Damian, Carina Wattmo, Lucrezia Hausner, Roger Bullock, Peter J Connelly, Bruno Dubois, Maria Eriksdotter, Michael Ewers, Elmar Graessel, Milica G Kramberger, Emma Law, Patrizia Mecocci, José L Molinuevo, Louise Nygård, Marcel GM Olde-Rikkert, Jean-Marc Orgogozo, Florence Pasquier, Karine Peres, Eric Salmon, Sietske AM Sikkes, Tomasz Sobow, René Spiegel, Magda Tsolaki, Bengt Winblad, Lutz Frölich

**Affiliations:** Network Aging Research, Heidelberg University, Bergheimer Str. 20, 69115 Heidelberg, Germany; Department of Geriatric Psychiatry, Central Institute of Mental Health, Medical Faculty Mannheim/Heidelberg University, Square J 5, 68159 Mannheim, Germany; Clinical Memory Research Unit, Department of Clinical Sciences, Lund University, 20502 Malmö, Sweden; Kingshill Research Centre, Victoria Hospital, 53 Downs Way, Swindon, SN3 6BW UK; Hon Senior Lecturer in Psychiatry at the University of Dundee, Murray Royal Hospital, Perth, PH2 7BH UK; Centre des Maladies Cognitives et Comportementales (IM2A), Institut du Cerveau et de la Moelle épinière (ICM), UMR-S975, Université Pierre et Marie Curie- Paris6, AP-HP, Hôpital de la Salpêtrière, 47 boulevard de l’Hôpital, 75013 Paris, France; Department of Neurobiology, Care Sciences and Society (NVS), Karolinska Institutet, Alfred Nobels allé 23, 14183 Huddinge, Sweden; Institute of Health and Nursing Science, Charité Center 1 for Health and Human Sciences, Augustenburger Platz 1, 13353 Berlin, Germany; Center for Health Services Research in Medicine, Department of Psychiatry and Psychotherapy, Friedrich-Alexander-University Erlangen-Nuremberg, Schwabachanlage 6, 91054 Erlangen, Germany; Department of Neurology, Centre for Cognitive Impairments, University Medical Centre Ljubljana, Zaloška cesta 2, 1000 Ljubljana, Slovenia; Scottish Dementia Clinical Research Network, Murray Royal Hospital, Perth, PH2 7BH UK; Institute of Gerontology and Geriatrics, University of Perugia, via Brunamonti 51, 06122 Perugia, Italy; Alzheimer’s Disease and Other Cognitive Disorders Unit, ICN, Hospital Clínic i Universitari, IDIBAPS, Villarroel 170, Barcelona, 08036 Spain; Division of Occupational Therapy, Department of Neurobiology, Care Sciences and Society (NVS), Karolinska Institutet, Fack 23200, 14183 Huddinge, Sweden; Department of Geriatrics, Radboud University Nijmegen Medical Centre, Reinier Postlaan 4, 6525 GC Nijmegen, the Netherlands; Department of Clinical Neurosciences, University Hospital Pellegrin, Place Amélie Raba-Léon, 33000 Bordeaux, France; INSERM U1171, CHU, Memory Clinic, University of Lille, rue Emile Laine, 59037 Lille, France; University of Bordeaux, ISPED, Centre INSERM U897-Epidemiologie-Biostatistique, 33000 Bordeaux, France; INSERM, ISPED, Centre INSERM U897-Epidemiologie-Biostatistique, 33000 Bordeaux, France; Memory Clinic, Department of Neurology, University of Liège, allée du 6 Août 8, 4000 Liège, Belgium; Alzheimer Center and Department of Epidemiology and Biostatistics, VU University Medical Center, De Boelelaan 1117, 1081 HV Amsterdam, the Netherlands; Department of Medical Psychology, Medical University of Lodz, 5 Sterling St, 90-425 Lodz, Poland; Memory Clinic, University Center for Medicine of Aging Basel, Felix Platter Hospital, Schanzenstr. 55, CH-4031 Basel, Switzerland; 3rd Department of Neurology, Aristotle University, Despere 3, Thessaloniki, 54621 Greece; Division of Neurogeriatrics, Department of Neurobiology, Care Sciences and Society (NVS), Center for Alzheimer Research, Karolinska Institutet, 14157 Huddinge, Sweden

## Abstract

**Introduction:**

There is a growing body of evidence that subtle deficits in instrumental activities of daily living (IADL) may be present in mild cognitive impairment (MCI). However, it is not clear if there are IADL domains that are consistently affected across patients with MCI. In this systematic review, therefore, we aimed to summarize research results regarding the performance of MCI patients in specific IADL (sub)domains compared with persons who are cognitively normal and/or patients with dementia.

**Methods:**

The databases PsycINFO, PubMed and Web of Science were searched for relevant literature in December 2013. Publications from 1999 onward were considered for inclusion. Altogether, 497 articles were retrieved. Reference lists of selected articles were searched for potentially relevant articles. After screening the abstracts of these 497 articles, 37 articles were included in this review.

**Results:**

In 35 studies, IADL deficits (such as problems with medication intake, telephone use, keeping appointments, finding things at home and using everyday technology) were documented in patients with MCI. Financial capacity in patients with MCI was affected in the majority of studies. Effect sizes for group differences between patients with MCI and healthy controls were predominantly moderate to large. Performance-based instruments showed slight advantages (in terms of effect sizes) in detecting group differences in IADL functioning between patients with MCI, patients with Alzheimer’s disease and healthy controls.

**Conclusion:**

IADL requiring higher neuropsychological functioning seem to be most severely affected in patients with MCI. A reliable identification of such deficits is necessary, as patients with MCI with IADL deficits seem to have a higher risk of converting to dementia than patients with MCI without IADL deficits. The use of assessment tools specifically designed and validated for patients with MCI is therefore strongly recommended. Furthermore, the development of performance-based assessment instruments should be intensified, as they allow a valid and reliable assessment of subtle IADL deficits in MCI, even if a proxy is not available. Another important point to consider when designing new scales is the inclusion of technology-associated IADL. Novel instruments for clinical practice should be time-efficient and easy to administer.

## Introduction

Mild cognitive impairment (MCI) is a controversial clinical entity, initially conceptualized as a transitional zone between normal aging and dementia. The most commonly used criteria for MCI—also known as Mayo criteria—were proposed by Petersen *et al*. [1,2]. These criteria require (1) a memory complaint, (2) normal activities of daily living, (3) normal general cognitive function, (4) abnormal memory for age and (5) absence of dementia. These criteria have been modified to expand the original MCI concept, including impairments in cognitive domains other than memory. Thus, the clinical phenotypes of amnestic MCI and nonamnestic MCI have been developed, which can both be further classified as single-domain or multiple-domain [3]. Discussion about the MCI criteria and their operationalization is ongoing [4], as the criteria neither specify methods to assess cognitive or functional capacity nor provide cutoff points for cognitive or functional scales to differentiate MCI from mild dementia.

Another important point of discussion is the existence of deficits in activities of daily living (ADL). ADL are divided into basic activities of daily living (BADL) and instrumental activities of daily living (IADL). BADL include self-maintenance skills such as bathing, getting dressed or eating, and IADL consist of more complex activities such as using public transportation, managing finances, or shopping [5]. The assessment of ADL is usually done by using rating scales, which are administered either to the patient or a proxy. Controversy exists about the ability of patients with MCI to adequately rate themselves, as they lack awareness of IADL deficits and overestimate their functional capacity [6-8]. Farias *et al*., however, reported no lack of awareness in patients with MCI compared with healthy controls [9]. There is evidence that proxies are not always a reliable source of information, as they have a tendency to over- or underestimate IADL deficits [8,10,11]. In some cases, a proxy is not available or has massive knowledge gaps. Direct measures requiring the patient to solve specific IADL-related tasks have better validity and do not have reporter bias. However, they allow observation of only a small excerpt of real-world performance and are quite time-consuming.

It is assumed that IADL require more complex neuropsychological processing capacity than BADL and therefore are more prone to deterioration triggered by cognitive decline [12,13]. Functional deficits have been observed early in the course of decline [14-16]. In an analysis of studies with a focus on BADL and IADL in subjects with MCI, dementia or no cognitive deficits, Nygård [17] suggested that IADL can be impaired before the onset of dementia and should therefore be included in the diagnosis of MCI.

These findings were taken into account by Winblad *et al*. [18], who proposed the following criteria for MCI: (1) not normal, not demented; (2) cognitive decline; and (3) preserved BADL and/or minimal impairment in complex instrumental functions. Thus, the criterion of “normal activities of daily living” has been revised to a less stringent one allowing for discrete IADL deficits in patients with MCI.

Over the last 15 years, a large amount of research has been conducted on IADL deficits in MCI. The aim of the present review is to summarize research results regarding the performance of patients with MCI in specific IADL (sub)domains compared with persons who are cognitively normal and/or patients with dementia. In addition, sample characteristics and applied IADL assessment methods—performance-based instruments versus self- and/or informant-reported questionnaires or interviews—are investigated.

## Methods

### Data sources

To identify relevant published papers, the electronic databases PubMed, Web of Science and PsycINFO were searched in December 2013. Publication dates were set from January 1999 to December 2013. This restriction was chosen to identify only papers that were published after the introduction of Petersen’s MCI definition [2]. The search terms “mild cognitive impairment” (MeSH term) or “MCI” were used in combination with the terms “activities of daily living” (MeSH term) or “ADL” or “instrumental activities of daily living” or “IADL” or “everyday functioning” or “functional ability” or “functional capability” or “functional deficits” or “functional impairment.” After removal of duplicates, 497 articles were retrieved from the 3 searched databases.

### Selection criteria

Titles and abstracts of the retrieved articles were screened by two authors (KJ and MD) independently and were rated to assess their relevance to the research question. If inconsistencies occurred, a third author (LH) was consulted. The following selection criteria were applied. (1) The abstract indicated that the focus of the study was the investigation of IADL in MCI versus healthy controls and/or dementia patients. (2) General IADL and/or specific subdomains were investigated. (3) The method of IADL assessment was standardized. (4) MCI was defined according to Petersen and/or Winblad criteria [2,3,18]. (5) No other concepts, such as cognitive impairment, no dementia [19,20], aging-associated cognitive decline [21] or age-associated memory impairment [22], were used. (6) The original article was written in English.

Articles that met the outlined criteria were included in the present review. Reference lists of the selected articles were searched to retrieve further relevant articles. Effect sizes (Cohen’s *d*) were calculated to allow a better evaluation of clinical relevance.

## Results

In total, 34 of the 497 papers were selected for review. Owing to the broad focus of the search terms to ensure retrieval of all relevant articles, the majority of articles did not meet the inclusion criteria (that is, no definition of MCI criteria, use of concepts other than Petersen and/or Winblad criteria). A further three articles were selected from among the reference lists of the selected papers. Thus, the content of the present review is formed from a total of 37 articles.

### Mild cognitive impairment sample characteristics

For the diagnosis of MCI, the criteria of Petersen or Winblad were applied across studies; their operationalization, however, varied. One-third of the studies used the original Petersen criteria supplemented by cutoffs on specific neuropsychological tests [15,23-34]. In the remaining studies, the use of the original clinical criteria published by Petersen *et al*. [2] was reported without specific cutoff values or with a combination of Petersen and Winblad criteria. Mean Mini Mental State Examination (MMSE) [35] scores ranged from 23.1 [36] to 28.7 points [37] for MCI samples, from 26.5 [36] to 29.4 points [30,38] for normal control samples and from 16.4 [39] to 25.5 points [40] for Alzheimer’s disease (AD) samples. In each examined study, however, the MMSE score for the MCI group was lower than that for the comparative control group and higher than that for the dementia sample.

### Study types and/or designs

The majority of the reported studies followed a cross-sectional design (29 studies [15,23-26,29,30,33,34,36-38,40-56]), and eight studies applied a longitudinal design [27,28,32,57-61]. In five of the longitudinal studies, risk of conversion to AD depending on IADL impairment was also assessed [27,28,32,58,60].

### Assessment instruments used

Altogether, 31 different instruments were used to assess IADL in patients with MCI (see Table 1 for details), including performance-based instruments, self- and informant-report rating questionnaires, and structured interviews. Of the 37 studies, 15 relied solely on informant-report rating questionnaires [23,28,29,31,33,40-43,45-48,54,58], 10 relied solely on performance-based assessments [24,26,30,32,38,50-53,57] and 6 relied solely on self-report rating instruments [27,36,55,56,59,61]. Three studies used both informant-report questionnaires and performance-based assessments [25,34,60]. Interestingly (and inconsistently), in three studies [15,25,44], the IADL of patients with MCI were rated by informants, whereas normal control subjects rated their IADL functioning themselves.

**Table 1:** **Instruments used for instrumental activities of daily living assessment** ^**a**^

**Abbreviation**	**Full instrument name**	**Type**	**IADL domains**	**Psychometric properties**
*Performance-based assessment instruments*				
DAFS [62]	Direct Assessment of Functional Status	P	6 domains: time orientation, communication, financial skills, shopping, grooming, eating	Good interrater and test–retest reliability, good evidence of discriminant and convergent validity, ceiling effects for time orientation, identify change and shopping
DOT [34]	Day-Out Task	P	8 tasks to prepare a day out (including packing a picnic basket, planning a bus route, gathering correct change for bus ride)	Interrater reliability: 96.92% agreement
EPT [63]	Everyday Problems Test	P	Problem solving related to medication use, meal preparation, telephone use, shopping, financial management, household management, transportation	Test–retest reliability: *r* = 0.93, internal consistency (Cronbach’s α) = 0.88. Validity: significant correlations with direct observation of older adults’ performance of everyday tasks (*r* = 0.67), older adults’ self-reports (*r* = 0.23) and dementia patients’ self-reports (*r* = 0.36)
FCI [64]	Financial Capacity Instrument	P	7 domains: basic monetary skills, financial conceptual knowledge, cash transactions, checkbook management, bank statement management, financial judgment, bill payment	For all subdomains: test–retest reliability *r* > 0.8, internal consistency (Cronbach’s α) > 0.8
META [53]	Management of Everyday Technology Assessment	P	10 technology-related items (including performing actions in a logical sequence, turning a button)	Acceptable person response validity
TFLS [65]	Texas Functional Living Scale	P	5 domains: time/orientation, money, communication, dressing, memory	Test–retest reliability: *r* = 0.93 in AD sample, test–retest reliability in control group: *r* = 0.52, strong correlation with MMSE scores (*r* = 0.92)
TIADL [66]	Timed Instrumental Activities of Daily Living	P	5 domains: shopping, finances, medication, telephone use, locating information on food labels (speed and accuracy)	Test–retest reliability: *r* = 0.85
UAB-DA [67]	University of Alabama at Birmingham Driving Assessment	P	Real-world, standardized route: lane control, gap judgment, turning, maintaining proper speed, stopping distance, signaling, obeying traffic signs, preturn and postturn position, spacing, steer steadiness, precrossing and postcrossing position, and proper scanning of driving space	Not reported
UCSD-UPSA [68]	University of California San Diego Performance-Based Skills Assessment	P	5 domains: household chores, communication, finances, transportation, planning recreational activities	Test–retest reliability: *r* = 0.92
VAPS [52]	Virtual Action Planning Supermarket	P	Virtual reality supermarket, 8 parameters: total distance, total time in seconds, number of items purchased, number of correct actions, number of incorrect actions, number of pauses, combined duration of pauses, time to pay	Validity (correlations between VAPS performance and executive functions): *r* = −0.40 to *r* = −0.63
*Self-report and informant-report rating instruments*				
ADCS-ADL [69]	Alzheimer’s Disease Cooperative Study/Activities of Daily Living Inventory	I	23 items (including shopping, hobbies, personal appliances; both IADL and BADL)	Moderate to good retest reliability, floor effects for financial abilities in individuals with dementia
ADCS-MCI-ADL-18 [69]	18-item Alzheimer’s Disease Cooperative Study/Activities of Daily Living Inventory adapted for patients with mild cognitive impairment	I	18 items (including shopping, hobbies, personal appliances; both IADL and BADL)	Not reported
ADCS-MCI-ADL-24 [45]	24-item Alzheimer’s Disease Cooperative Study/Activities of Daily Living scale adapted for patients with mild cognitive impairment	I	24 items (original ADCS-MCI-ADL scale plus 6 MCI-specific items, including driving a car, organizing medication)	Not reported
ADL-PI [70]	Activities of Daily Living-Prevention Instrument	I	15 items (including completing and/or organizing activities, taking medication, using telephone, finding belongings, managing finances)	Retest reliability: from *r* = 0.69 to *r* = 0.74
Bayer-ADL [71]	Bayer Activities of Daily Living Scale	I	25 items (2 BADL items, 18 specific IADL items, 5 items for cognitive functions)	Internal consistency (Cronbach’s α > 0.98)
DAD [72]	Disability Assessment for Dementia	I	IADL part with 23 items (meal preparation, telephoning, going on an outing, finances, medication, housework, leisure) and BADL part with 17 items	Internal consistency (Cronbach’s α = 0.96), interrater reliability (ICC = 0.95), test–retest reliability (ICC = 0.96)
DAD-6 [40]	6-item Disability Assessment for Dementia	I	6 items: meal preparation, telephoning, going on an outing, handling finances and correspondence, medication, leisure, housework	Not reported
DHQ [59]	Driving Habits Questionnaire	S	Driving difficulty in 8 different situations and driving frequency	Retest reliability: from *r* = 0.65 to *r* = 0.86 for the 8 situations
ETUQ [56]	Everyday Technology Use Questionnaire	S	86 items (including questions about technology at home and outside, communication)	Acceptable levels of internal scale validity, unidimensionality, and person response validity
FAQ [73]	Functional Activities Questionnaire	S/I	10 items (including finances, shopping, remembering appointments, playing games, preparing a meal, traveling, remembering appointments)	Not reported
FC-ADL [74]	Functional Capacities for Activities of Daily Living	I	50 statements reflecting possible IADL difficulties	Not reported
4-IADL [27]	4 IADL scale items chosen from Lawton and Brody’s Instrumental Activities of Daily Living [5]	S	4 items: telephone use, finances, medication, transportation	Not reported
9-IADL [58]	9-item IADL scale	I	9 items: medication responsibility, ability to buy food, to prepare meals, to keep the home clean, to use the telephone, to handle finances, to use public transportation, to orientate oneself outside, to visit people	Not reported
IQCODE [75]	Informant Questionnaire on Cognitive Decline in the Elderly	I	26 items (including finances, communication, memory, household appliances)	Cronbach’s α = 0.96, correlation with MMSE (*r* = 0.74)
KI-IADL [34]	Knowledgeable Informant report about Instrumental Activities of Daily Living	I	50 questions assessing 10 IADL domains: using the phone, traveling, shopping, preparing meals, household activities, conversation, organization, social functioning, medication management, financial management	Not reported
L&B IADL [5]	Lawton and Brody’s Instrumental Activities of Daily Living	S/I	8 items: shopping, grooming, medication responsibility, handling finances, mode of transportation, telephone use, food preparation, telephone use	Interrater correlation: *r* = 0.85
ROIL [76]	Record of Independent Living	I	37 items assessing 3 domains: activities, communication, behavior	Not reported
SR-IADL [77]	Self-report Instrumental Activities of Daily Living	S	Items include handling money, keeping appointments, planning meals (IADL performance and difficulty)	Reliability: *r* = 0.74
S-IADL [78]	Seoul-Instrumental Activities of Daily Living	S/I	15 items (including ability to prepare a balanced meal, remember appointments, ability to keep financial records, remember to take medication)	Good reliability and validity
SIB-R [79]	Scales of Independent Behavior–Revised	S/I	13 subscales organized into 4 adaptive behavior clusters: (1) social interaction and communication, (2) personal living, (3) community living, (4) motor skills	Self-report: internal consistency (Cronbach’s α) = 0.92, test–retest reliability: *r* = 0.80
				Informant-report: internal consistency (Cronbach’s α) = 0.95, test–retest reliability: *r* = 0.84
T-ADLQ [54]	Technology–Activities of Daily Living Questionnaire	I	7 subscales (self-care, household care, employment and recreation, shopping and money, travel, communication, technology)	Cronbach’s α = 0.86; validity: significant correlations with the MMSE (*r* = −0.70)

^a^AD, Alzheimer’s disease; ADL, Activities of daily living; BADL, Basic activities of daily living; I, Informant-report; IADL, Instrumental activities of daily living; ICC, Intraclass correlation coefficient; MMSE, Mini Mental State Examination; P, Performance-based; S, Self-report.

### Mild cognitive impairment subtypes

According to Petersen *et al*. [1], MCI has two major subtypes: amnestic and nonamnestic. Both can be further divided into single-domain and multidomain types. Among the 37 studies included in this review, IADL performance was analyzed between MCI subtypes in 8 studies [23,31,33,37,40,48,58,61].

### Instrumental activities of living in patients with mild cognitive impairment

Among the 37 studies included in this review, all but 2 studies [38,42] found IADL deficits in patients with MCI compared with control subjects without cognitive impairment on at least one applied instrument. In the following sections, we first report results of studies investigating global IADL (see Table 2), then results of studies in which informant-report measures were used and studies using self-report measures (see Table 3).

**Table 2:** **Studies investigating global instrumental activities of daily living functioning** ^**a**^

**Author**	**Year**	**MCI criteria**	**Number of subjects**	**Mean age, yr (SD)**	**Mean MMSE score (SD)**	**IADL measures used**	**Results and effect sizes (Cohen’s** ***d*** **)**
*Performance-based instruments*							
Binegar *et al*. [57]	2009	Petersen	30 MCI	MCI 72.8 (7.9)	MCI 27.3 (2.2)	TFLS	Total score: MCI < NC (*d* = 0.61); subscales: significant for memory subscale (*d* = 0.85), but not for time/orientation, money, communication, dressing
		Clinical	30 NC	NC 73.7 (6.9)	NC 29.2 (1.0)		
				ns	significant		
Giovannetti *et al*. [24]	2008	Petersen	25 MCI	MCI 72.2 (6.7)	MCI 27.6 (1.4)	NAT	Total score: NC > MCI > AD; MCI versus NC: *d* = 1.05, MCI versus AD: *d* = 1.46 Error score: NC < MCI < AD; MCI versus NC: *d* = 0.74, MCI versus AD: *d* = 1.78
		1.5 SD below	18 NC	NC 73.1 (3.2)	NC 28.5 (1.0)		
		MMSE ≥25	25 mild AD	AD 73.6 (3.8)	AD 22.4 (2.8)		
				ns	(NC = MCI) > AD, *P* < 0.05		
Goldberg *et al*. [25]	2010	Petersen	26 MCI	MCI 77.5 (7.1)	MCI 26.1 (2.3)	UCSD-UPSA	UCSD-UPSA: NC > MCI > AD; MCI versus NC: *d* = 0.86, MCI versus AD: *d* = 1.81 ADCS-ADL: (NC = MCI ) > AD; MCI versus AD: *d* = 1.81
		1.5 SD below	50 NC	NC 68.8 (9.9)	NC 28.5 (1.5)	Additional informant-report: ADCS-ADL (NC: self-report)	
		CDR 0.5	22 AD	AD 78.4 (5.4)	AD 20.3 (3.4)		
		MMSE ≥24					
Pereira [60]	2010	Petersen	31 MCI	MCI 72.6 (7.0)	MCI 27.3 (2.3)	DAFS	DAFS total score NC > MCI > AD; MCI versus NC: *d* = 1.58, MCI versus AD: *d* = 2.18 DAFS subdomains: NC > MCI for finances and shopping, but not time orientation, communication, grooming, eating, which were worse only in AD; IQCODE total score: NC > MCI > AD; MCI versus NC: *d* = 1.00, MCI versus AD: *d* = 0.77
		Clinical	32 NC	NC 71.6 (5.6)	NC 28.8 (1.5)		
			26 AD	AD 77.9 (6.0)	AD 19.5 (5.5)	Additional informant-report: IQCODE	
				AD > (MCI/NC)	AD < (MCI = NC)		
Schmitter-Edgecombe *et al*. [34]	2012	Petersen	38 MCI	MCI 70.5 (8.6)	Not reported	DOT	DOT: MCI < NC for completion time (*d* = 0.60) and accuracy (*d* = 0.61)
		1.5 SD below	38 NC	NC 69.3 (7.9)		Additional informant-report: KI-ADL	KI-ADL: MCI < NC (*d* = 0.50)
				ns			
Wadley *et al*. [50]	2008	Petersen	50 MCI	MCI 70.0 (7.9)	Not reported	Timed IADL	MCI = NC for accuracy
		Clinical	59 NC	NC 67.8 (7.1)			MCI < NC for speed (*d* = 0.75), significant subdomains telephone (*d* = 0.56), grocery (*d* = 0.75), medication (*d* = 0.51), nutrition information (*d* = 0.52)
				ns			
*Informant-report rating instruments*							
Ahn *et al*. [41].	2009	Petersen/Winblad	66 MCI	MCI 70.8 (7.3)	MCI 24.8 (3.1)	Seoul-IADL	MCI < NC (*d* = 1.62)
		1.5 SD below	61 NC	NC 64.4 (5.6)	NC 27.6 (1.4)		
		CDR 0.5		significant			
Boeve *et al*. [42]	2003	Petersen	13 MCI	MCI 94.3 (2.6)	MCI 26.8 (1.6)	ROIL	MCI = NC, MCI > dementia (*d* = 2.93)
		Clinical	56 NC	NC 93.8 (2.5)	NC 27.9 (2.3)		
			42 Dementia	Dementia 94.8 (2.6)	Dementia 18.6 (5.0)		
				ns	AD < (MCI = NC)		
Brown *et al*. [15]	2011	Petersen	394 MCI	MCI 74.9 (7.4)	MCI 27.0 (1.8)	FAQ (NC: self-report)	Severity of deficits: NC > MCI > AD; MCI versus NC: *d* = 1.04, MCI versus AD: *d* = 1.71 Number of deficits: NC < MCI < AD; MCI versus NC: *d* = 1.28, MCI versus AD: *d* = 1.62
		1.5 SD below	229 NC	NC 75.9 (5.0)	NC 29.1 (1.0)		
		CDR 0.5	193 AD	AD 75.3 (7.5)	AD 23.3 (2.1)		
		MMSE ≥24		ns	significant		
Jefferson *et al*. [43]	2008	Petersen/Winblad	38 MCI	MCI 74.6 (7.5)	MCI 28.0 (1.7)	L&B IADL	L&B IADL: MCI = NC, FC-ADL: MCI < NC (*d* = 0.84)
		Clinical	39 NC	NCI 72.4 (5.5)	NC 29.3 (0.9)	FC-ADL	
				ns	significant		
Mariani *et al*. [44]	2008	Petersen/Winblad	132 MCI	MCI 76.1 (5.8)	MCI 25.7 (1.6)	L&B IADL (MCI: informant-report, NC: self-report)	MCI < NC (*d* = 0.29)
		below normality cutoff	249 NC	NC 72.2 (7.5)	NC 28.1 (1.2)		
				significant	significant		
Pedrosa *et al*. [45]	2010	Petersen/Winblad	30 MCI	MCI 75.7 (6.4)	MCI 24.4 (3.3)	ADCS-MCI-ADL-18 ADCS-MCI-ADL-24 L&B-IADL	ADCS-MCI-ADL-18: NC > MCI > AD; MCI versus NC: *d* = 1.39, MCI versus AD: *d* = 2.27 ADCS-MCI-ADL-24: NC > MCI > AD; MCI versus NC: *d* = 1.67, MCI versus AD: *d* = 2.33 L&B IADL: NC > MCI > AD; MCI versus NC: *d* = 2.0, MCI versus AD: *d* = 2.89
		1 SD below	31 NC	NC 72.2 (8.0)	NC 27.7 (3.0)		
			33 AD	AD 76.1 (7.5)	AD 16.5 (5.2)		
Perneczky *et al*. [47]	2006	Petersen/Winblad	48 MCI	MCI 69.2 (8.3)	MCI 26.5 (2.3)	ADCS-MCI-ADL-18 Bayer-ADL IQCODE	ADCS-MCI-ADL-18: MCI < NC (*d* = 1.98) Bayer-ADL: MCI < NC (*d* = 1.95) IQCODE: MCI < NC (*d* = 1.09)
		1 SD below	42 NC	NC 66.7 (9.3)	NC 29.3 (0.7)		
		CDR 0.5		ns	significant		
Perneczky *et al*. [46]	2006	Petersen/Winblad	45 MCI	MCI 69.2 (8.3)	MCI 26.9 (1.4)	ADCS-MCI-ADL-18 Bayer-ADL	ADCS-MCI-ADL-18: MCI < NC (*d* = 1.89) Bayer-ADL: MCI < NC (*d* = 2.44)
		1 SD below	30 NC	NC 66.7 (9.3)	NC 29.3 (0.7)		
		CDR 0.5		ns			
Reppermund *et al*. [29]	2011	Petersen	293 MCI	MCI 78.8 (4.7)	MCI 28.0 (1.5)	Bayer-ADL	Bayer-ADL total: MCI < NC (*d* = 0.32)
		1.5 SD below	469 NC	NC 78.3 (4.7)	NC 28.8 (1.2)		Bayer-ADL high cognitive demand: MCI < NC (*d* = 0.40)
				ns			
							Bayer-ADL low cognitive demand: MCI = NC
Reppermund *et al*. [28]	2013	Petersen	227 MCI	MCI 78.6 (4.4)	MCI 28.3 (1.4)	Bayer-ADL	Bayer-ADL total: MCI < NC (*d* = 0.39)
		1.5 SD below	375 NC	NC 77.9 (4.6)	NC 28.9 (1.2)		Bayer-ADL high cognitive demand: MCI < NC (*d* = 0.40) Bayer-ADL low cognitive demand: MCI < NC (*d* = 0.27), IADL performance at baseline predicted conversion to dementia at 2-year follow-up
				ns	significant		
*Self-report rating instruments*							
Kim *et al*. [36]	2009	Winblad	255 MCI	MCI 72.0 (6.0)	MCI 23.1 (4.5)	Seoul-IADL	MCI < NC (*d* = 0.27)
		1 SD below	311 NC	NC 70.7 (6.0)	NC 26.5 (3.3)		
				significant	significant		
Peres *et al*. [27]	2006	Petersen	285 MCI	Total sample: 80.8 (5.6)	Not reported	4-IADL	NC > MCI > dementia
		1.5 SD below	828 NC				
			149 dementia				
*Comparison of MCI subtypes: informant-report rating instruments*							
Aretouli *et al*. [23]	2010	Petersen	124 MCI	MCI 76.3 (7.5)	MCI 28.2 (1.3)	ADL-PI IQCODE	ADL-PI: MCI < NC, *P* < 0.001; all MCI subgroups < NC, *P* < 0.001, md = sd; am = nonam IQCODE: MCI < NC, *P* < 0.001; true for all subgroups; multiple > single, am = nonam
		1.5 SD below	(36 asMCI	NC 72.4 (7.3)	NC 29.3 (0.9)		
		CDR 0.5	45 amMCI	significant	significant		
			26 nasMCI				
			17 namMCI)				
			68 NC				
Luck *et al*. [58]	2011	Winblad	161 MCI	MCI 81.9 (5.0)	Not reported	9 IADL items (Schneekloth and Potthoff [80])	MCI < NC (aMCI = naMCI; aMCI < NC (*d* = 0.17), naMCI = NC) MCI + IADL deficits: higher risk of conversion to dementia MCI + IADL: 47.4% versus MCI-IADL: 31.4%; NC + IADL: 26.7% versus NC-IADL: 8.0%
		1 SD below	(36 asMCI	(aMCI 81.6 (4.8),			
			42 amMCI	naMCI 82.2 (5.2))			
			60 nasMCI	NC 81.2 (4.7)			
			23 namMCI)	ns			
			723 NC				
de Rotrou [40]	2012	Petersen	53 MCI	MCI 78.6 (7.3)	MCI 26.2 (2.2)	DAD-6	NC > MCI > AD; MCI versus NC: *d* = 1.29, MCI versus AD: *d* = 1.66 NC > sdMCI (*d* = 1.59), sdMCI > mdMCI (*d* = 1.37)
		Clinical	(29 sdMCI	NC 80.9 (4.2)	NC 29.1 (1.0)		
			24mdMCI)	Dementia 80.6 (6.2)	Dementia 25.5 (1.8)		
			55 NC	ns	All significant		
			31 Dementia				
Tam *et al*. [48]	2007	Petersen/Winblad	54 asMCI	asMCI 79.3 (6.1)	asMCI 25.4 (3.0)	DAD	IADL subscale: (NC = asMCI) > amMCI > AD; amMCI versus NC: *d* = 0.98, asMCI versus amMCI: *d* = 0.80, asMCI versus AD: *d* = 2.93, amMCI versus AD: *d* = 1.71
		CDR 0.5	93 amMCI	amMCI 80.1 (6.5)	amMCI 22.3 (3.1)		
		1 SD below	78 NC	NC 77.1 (5.1)	NC 27.2 (2.1)		
			85 AD	AD 84.5 (5.9)	AD 17.9 (3.2)		
Teng *et al*. [31]	2010	Petersen	1108 MCI	as 77.0 (9.2)	as 27.8 (1.8)	FAQ	NC > asMCI/amMCI/nasMCI; asMCI = amMCI, nasMCI = namMCI
		MMSE ≥24	(532 asMCI	am 75.3 (8.5)	am 27.4 (1.8)		
			340 amMCI	nas 74.1 (8.6)	nas 28.2 (1.7)		
			162 nasMCI	nam 73.0 (6.8)	nam 27.8 (1.5)		
			74 namMCI)	NC 74.8 (9.1)	NC 29.0 (1.2)		
			3,036 NC	significant			
Yeh *et al*. [33]	2011	Petersen	56 asMCI	asMCI 77.5 (6.7)	asMCI 26.6 (1.6)	DAD	NC > MCI (as = am) > AD; asMCI versus NC: *d* = 0.9, amMCI versus NC: *d* = 1.06, asMCI versus AD: *d* = 2.23, amMCI versus AD: *d* = 1.9
		1 SD below	94 amMCI	amMCI 78.9 (5.8)	amMCI 25.8 (1.6)		
		MMSE ≥24	64 NC	NC 76.5 (6.6)	NC 28.5 (1.3)		
			102 AD	AD 79.6 (6.1)	AD 20.9 (3.1)		
*Comparison of MCI subtypes: self-report rating instruments*							
Wadley *et al*. [61]	2007	Petersen/Winblad	84 aMCI	aMCI 77.0 (7.0)	aMCI 26.0 (1.9)	IADL (Home Care questionnaire)	IADL performance: aMCI/mdMCI < NC, naMCI = NC; aMCI versus NC: *d* = 0.23, mdMCI versus NC: *d* = 0.31; aMCI < naMCI: *d* = 0.23 IADL difficulty: all MCI subgroups < NC; aMCI versus NC: *d* = 0.57, naMCI versus NC: *d* = 0.27, mdMCI versus NC: *d* = 0.57; aMCI < naMCI: *d* = 0.23
		1.5 SD below	171 naMCI	naMCI 76.5 (6.2)	naMCI 26.2 (2.1)		
			89 mdMCI	mdMCI 78.8 (6.6)	mdMCI 25.1 (1.8)		
			2,110 NC	NC 72.9 (5.4)	NC 27.6 (1.8)		
				significant			
*Comparison of MCI subtypes and all three types of instruments*							
Burton *et al*. [37]	2009	Petersen/Winblad	6 asMCI	asMCI 79.5 (5.7)	asMCI 26.8 (2.5)	Performance-based: EPT	Self-report: SIB-R: NC > mdMCI (*d* = 0.71), sdMCI > mdMCI (*d* = 0.45), L&B: MCI = NC; L&B IADL: MCI = NC Informant-report SIB-R: NC > sdMCI (*d* = 0.46), NC > mdMCI (*d* = 0.51); L&B IADL: MCI = NC EPT: NC > sdMCI > mdMCI; sdMCI versus NC: *d* = 0.50, sdMCI versus mdMCI: *d* = 1.54
		1 SD below	39 nasMCI	nasMCI 77.5 (5.6)	nasMCI 28.7 (1.3)	Self-report: L&B IADL, SIB-R; Informant-report: L&B IADL, SIB-R	
			19 amMCI	amMCI 82.0 (5.0)	amMCI 28.2 (1.3)		
			28 namMCI	namMCI 79.6 (4.9)	namMCI 28.7 (1.1)		
			158 NC	NC 73.6 (4.7)	NC 28.9 (1.2)		

^a^AD, Alzheimer’s disease; ADCS-ADL, Alzheimer’s Disease Cooperative Study/Activities of Daily Living Inventory; ADCS-MCI-ADL-18, 18-item Alzheimer’s Disease Cooperative Study/Activities of Daily Living Inventory adapted for patients with mild cognitive impairment; ADCS-MCI-ADL-24, 24-item Alzheimer’s Disease Cooperative Study/Activities of Daily Living Inventory adapted for patients with mild cognitive impairment; ADL, Activities of daily living; ADL-PI, Activities of Daily Living-Prevention Instrument; am, Amnestic multiple domain; aMCI, Amnestic mild cognitive impairment; as, Amnestic single domain; BADL, Basic activities of daily living; Bayer-ADL, Bayer Activities of Daily Living Scale; CDR, Clinical dementia rating; DAD, Disability Assessment for Dementia; DAD-6, 6-item Disability Assessment for Dementia; DAFS, Direct Assessment of Functional Status; DHQ, Driving Habits Questionnaire; DOT, Day-Out Task; EPT, Everyday Problems Test; ETUQ, Everyday Technology Use Questionnaire; FAQ, Functional Activities Questionnaire; FC-ADL, Functional Capacities for Activities of Daily Living; FCI, Financial Capacity Instrument; FC-IADL, Functional Capacities for Instrumental Activities of Daily Living; IADL, Instrumental activities of daily living; 4-IADL, 4-item Instrumental Activities of Daily Living scale items chosen from Lawton and Brody; 9-IADL, 9-item Instrumental Activities of Daily Living scale; ICC, Intraclass correlation coefficient; IQCODE, Informant Questionnaire on Cognitive Decline in the Elderly; KI-IADL, Knowledgeable Informant report about Instrumental Activities of Daily Living; L&B IADL, Lawton and Brody’s Instrumental Activities of Daily Living; MCI, Mild cognitive impairment; md, Multiple domain; META, Management of Everyday Technology Assessment; MMSE, Mini Mental State Examination; nam, Nonamnestic multiple domain; naMCI, Nonamnestic mild cognitive impairment; nas, Nonamnestic single domain; NAT, Naturalistic action task; NC, Normal control; NIA-AA, National Institute on Aging and Alzheimer’s Association; ns, nonsignificant; ROIL, Record of Independent Living; sd, Single domain; SD, Standard deviation; S-IADL, Seoul-Instrumental Activities of Daily Living; SIB-R, Scales of Independent Behavior–Revised; SR-IADL, Self-report Instrumental Activities of Daily Living; TADL-Q, Technology–Activities of Daily Living Questionnaire; TFLS, Texas Functional Living Scale; TIADL, Timed Instrumental Activities of Daily Living; UAB-DA, University of Alabama at Birmingham Driving Assessment; UCSD-UPSA, University of California, San Diego Performance-Based Skills Assessment; VAPS, Virtual Action Planning Supermarket.

**Table 3:** **Studies investigating specific instrumental activities of daily living domains** ^**a**^

**Author**	**Year**	**MCI criteria**	**Number of subjects**	**Mean age, yr (SD)**	**Mean MMSE score (SD)**	**IADL measures**	**Results and effect sizes (Cohen’s** ***d*** **)**
*Financial capacity: performance-based instruments*							
Griffith *et al*. [26]	2003	Petersen	21 MCI	MCI 68.1 (8.8)	MCI 28.4 (1.2)	FCI	NC > MCI > AD; MCI versus NC: *d* = 1.14, MCI versus AD *d* = 1.21
		CDR 0.5	21 NC	NC 66.7 (7.2)	NC 29.3 (1.0)		
			22 AD	AD 71.5 (9.2), ns	AD 24.1 (2.6)		
Sherod *et al*. [30]	2009	Petersen	113 MCI	MCI 70.3 (7.4)	MCI 28.1 (1.9)	FCI	NC > MCI > AD; MCI versus NC: *d* = 1.03, MCI versus AD: *d* = 0.87
		1.5 SD below	85 NC	NC 67.2 (8.2)	NC 29.4 (0.9)		
			43 AD	AD 73.8 (8.5)	AD 24.6 (2.9)		
				all significant			
Triebel *et al*. [32]	2009	Petersen	87 MCI	ADcon 74.4 (6.0)	ADcon 27.0 (1.9)	FCI	NC > MCI; ADnon versus NC: *d* = 0.83, ADcon versus NC: *d* = 1.83
		1.5 SD below	(25 ADcon, 62 ADnon)	ADnon 68.5 (7.5)	ADnon 28.6 (1.4)		
			76 NC	NC 66.7 (8.5)	NC 29.4 (1.0)		
*Management of everyday technology: performance-based instruments*							
Malinowsky *et al*. [53]	2010	Petersen	33 MCI	MCI 70.5 (8.4)	MCI 27.5 (1.9)	META	NC > MCI > AD, MCI versus NC: *d* = 0.66, MCI versus AD: *d* = 1.23
			45 NC	NC 73.2 (9.7)	NC 29.3 (1.1)		
			38 AD	AD 75.3 (9.1)	AD 23.5 (3.3)		
Malinowsky *et al*. [38]	2012	Petersen/Winblad	33 MCI	MCI 70.8 (8.6)	MCI 27.5 (1.9)	META	NC > AD, MCI = NC
			42 NC	NC 72.6 (9.7)	NC 29.4 (1.0)		
			35 AD	AD 75.5 (9.2)	AD 23.5 (3.4)		
				ns			
*Management of everyday technology: informant-report rating instruments*							
Munoz-Neira *et al*. [54]	2012	Winblad	21 MCI	MCI 71.3 (9.1)	MCI 26.1 (2.5)	T-ADLQ	Total score: NC > MCI > AD, MCI versus NC: *d* = 0.62, MCI versus AD: *d* = 1.47 Subscales: NC > MCI on 2 subscales: employment and recreation: *d* = 0.54, travel: *d* = 0.55
			44 NC	NC 74.1 (7.3)	NC 27.8 (2.3)		
			63 AD	AD 73.9 (8.7)	AD 17.9 (5.8)		
*Management of everyday technology: self-report rating instruments*							
Nygård *et al*. [55]	2011	Petersen/Winblad	37 MCI	MCI 67.0 (7.47)	MCI 27.5 (2.1)	ETUQ (support of proxy possible for patients with AD and MCI)	Perceived relevance of ET: NC > MCI > AD; MCI versus NC: *d* = 0.51, MCI versus AD: *d* = 1.26
			44 NC	NC 69.0 (9.58)	NC 29.1 (1.1)		
			37 AD	AD 72.0 (8.92)	AD 25.4 (2.8)		
				ns	ns		Perceived difficulty of ET: NC < MCI < AD; MCI versus NC: *d* = 0.82, MCI versus AD: *d* = 1.26
Rosenberg *et al*. [56]	2009	Petersen	30 MCI	MCI 74.0 (6.9)	MCI 27.0 (2.4)	ETUQ (support of proxy possible for patients with AD and MCI)	Perceived relevance of ET: NC > MCI = AD; MCI versus NC: *d* = 1.66
			93 NC	NC 74.0 (7.6)	NC 28.0 (1.7)		Perceived difficulty of ET: NC < MCI < AD; MCI versus NC: *d* = 0.59, MCI versus AD: *d* = 1.00
			34 AD	AD 73.0 (8.4)	AD 24.0 (3.3)		
				ns			
*Driving capacity: performance-based instruments*							
Wadley *et al*. [51]	2009	Petersen	46 MCI	MCI 71.3 (7.8)	Not reported	UAB-DA	MCI < NC, *d* = 0.46
			59 NC	NC 67.1 (6.7)			
				significant			
*Driving capacity: self-report rating instruments*							
O’Connor *et al*. [59]	2010	Petersen/Winblad	304 MCI	MCI 76.8 (6.5)	Not reported	DHQ	(aMCI = naMCI = mdMCI) < NC (driving frequency, driving difficulty, driving space) differed at baseline and faster rates of decline Driving frequency: aMCI versus NC: *d* = 0.31, naMCI versus NC: *d* = 0.24, mdMCI versus NC: *d* = 0.14 Driving difficulty: aMCI versus NC: *d* = 0.35, naMCI versus NC: *d* = 0.36, mdMCI versus NC: *d* = 0.45 Driving space: aMCI versus NC: *d* = 0.42, naMCI versus NC: *d* = 0.51, mdMCI versus NC: *d* = 0.43
		1.5 SD below	(82 aMCI	NC 72.6 (5.3)			
			140 naMCI	significant			
			82 mdMCI)				
			2,051 NC				
*Shopping capacity: performance-based instruments*							
Werner *et al*. [52]	2009	Petersen	30 MCI	MCI 69.3 (7.4)	MCI 27.5 (1.3)	VAPS	MCI < NC; significant subscales: distance *d* = 0.29, trajectory duration: *d* = 1.16, duration of pauses: *d* = 0.89
			30 NC	NC 69.6 (7.3)	NC 29.4 (0.7)		
				ns	significant		

^a^AD, Alzheimer’s disease; ADcon, Converters to Alzheimer’s disease; ADnon, Nonconverters to Alzheimer’s disease; aMCI, Amnestic mild cognitive impairment, both single and multiple domains; CDR, Clinical dementia rating; DHQ, Driving Habits Questionnaire; ETUQ, Everyday Technology Use Questionnaire; FCI, Financial Capacity Instrument; MCI, Mild cognitive impairment; mdMCI, Multiple-domain mild cognitive impairment; NC, Normal control; ns, Nonsignificant; UAB-DA, University of Alabama at Birmingham Driving Assessment; VAPS, Virtual Action Planning Supermarket.

### Global instrumental activities of daily living rating instruments

#### Performance-based instruments

Schmitter-Edgecombe *et al*. [34] designed the Day-Out Task (DOT), which requires multitasking in a real-world setting. Participants have to prepare for a day out and complete related tasks such as planning a bus route or packing specific items in a picnic basket. Patients with MCI required more time to complete the DOT than healthy controls and made more errors while solving the subtasks. By means of the Timed IADL, Wadley *et al*. [50] investigated both the speed and accuracy of patients with MCI in solving tasks related to shopping, finances, medication, telephone use and locating information on food labels. Patients with MCI took significantly longer than normal controls to solve the tasks and were less accurate. Using the Direct Assessment of Functional Status (DAFS), Pereira *et al*. [60] found that patients with MCI performed significantly worse than healthy controls and better than AD patients. Financial and shopping skills were the items that differentiated patients with MCI from healthy controls. Binegar *et al*. [57] applied the Texas Functional Living Scale and detected a significant but small difference between patients with MCI and controls. Interestingly, they mentioned that the performance of patients with MCI on this direct measure was much better (47 points) than that of patients with mild AD (31 points) in a previously conducted study [65].

Using the Naturalistic Action Task, Giovanetti *et al*. [24] found that patients with MCI performed significantly worse than healthy controls, but better than persons with mild AD, on all three assessed tasks: preparing toast and coffee, wrapping a gift and preparing a lunch box. When cutoff scores were applied, no controls, but 24% of the patients with MCI and 76% of the AD group, fell within the impaired range. Goldberg *et al*. found a similar pattern of results when they applied a novel performance-based assessment (the University of California San Diego Performance-Based Skills Assessment): The cognitively normal control group outperformed the MCI group, which in turn performed better than the mild to moderate AD group [25]. Interestingly, using the informant-report Alzheimer’s Disease Cooperative Study/Activities of Daily Living Inventory (ADCS-ADL), they detected no significant differences between patients with MCI and persons who were cognitively normal.

All of the performance-based instruments detected significant differences in IADL functioning between patients with MCI and healthy controls, as well as between patients with MCI and patients with dementia, respectively. Furthermore, patients with MCI needed more time to complete tasks than healthy controls and less time than patients with dementia. Calculated effect sizes were medium to large. In terms of effect sizes, the DAFS was the best measure for detecting differences in global IADL functioning between MCI and healthy controls (Cohen’s *d* = 1.58) and between MCI and AD (Cohen’s *d* = 2.18).

#### Informant-report rating instruments

Using the Seoul-IADL, Ahn *et al*. [41] found deficits in patients with MCI compared with healthy controls in the domains of telephone use, meal preparation, medication intake, management of belongings, keeping appointments, talking about recent events and performing leisure activities and/or hobbies. They concluded that IADL requiring memory or frontal cortex executive functioning are at particular risk of decline in MCI. Jefferson *et al*. [43] applied an error-based questionnaire of functional capacity (FC-IADL). The FC-IADL measures specific behaviors such as “getting lost in familiar places” and “does not use tools for the proposed use.” On this questionnaire, patients with MCI scored more than 1.5 standard deviations (SD) worse than normal controls. In contrast, no statistically or clinically significant differences were found for the informant-report Lawton and Brody IADL scale.

In contrast, two other studies applying Lawton and Brody’s IADL scale [44,45] showed that patients with MCI had deficits compared with controls regarding shopping, taking medications and handling finances.

Using the Record of Independent Living, Boeve *et al*. [42] found no significant differences between patients with MCI and healthy controls, but they did observe differences between patients with MCI and controls compared with dementia patients. This study is exceptional within this review because the participants were 90 to 100 years of age. Furthermore, the MCI group was very small (n = 13, compared with 56 healthy controls and 42 patients with dementia). Perneczky *et al*. [47] applied a questionnaire specifically designed for measuring IADL in MCI—the ADCS-MCI-ADL [69]—and found greater informant-reported impairments for the MCI group than among the age- and sex-matched cognitively normal controls. Pedrosa *et al*. [45] also reported better ADCS-MCI-ADL scores for healthy controls than for patients with MCI. Consistent observations—that is, differences between patients with MCI and healthy controls—in both studies were observed for finding personal belongings, balancing a checkbook, keeping appointments, using a telephone and talking about recent events. Furthermore, Pedrosa et al. compared the original ADCS-MCI-ADL scale with an extended version. (The authors added six items that they considered useful for MCI populations.) The 24-item version distinguished patients with MCI and healthy controls more reliably than the 18-item version [45]. Reppermund *et al*. [29], using the Bayer-ADL scale, found significant differences between patients with MCI and healthy controls. This effect was due to deficits of patients with MCI in the domains of observing important dates or events, reading, describing recent events, taking part in a conversation, taking a message, doing two tasks at a time, coping with unfamiliar situations and performing a task while under pressure. Conducting a factor analysis, the authors further subdivided the items into IADL with high or low cognitive demands. Group differences emerged only for the high cognitive demand factor, which consisted mainly of the items mentioned above, which in turn were responsible for the group differences between healthy controls and MCI subjects. The low cognitive demand factor consisted of items such as shopping, using transportation and preparing food. The same work group [28] gathered longitudinal data and again found differences in the Bayer-ADL scale between patients with MCI and healthy controls at baseline and at 2-year follow-up. For healthy controls, Bayer-ADL items with high cognitive demand predicted conversion to MCI and dementia at follow-up. Using the Functional Activities Questionnaire (FAQ), Brown *et al*. [15] detected significant differences between patients with MCI and healthy controls, and patients with MCI showed more deficits than healthy controls regarding financial skills and remembering events.

With the exception of one study [42], differences between patients with MCI and healthy controls were consistently detected. Deficits regarding financial abilities and memory-related IADL such as keeping appointments or remembering events were common themes across studies. With large effect sizes and consistent results across studies, the informant-reported ADCS-MCI-ADL seems to be a useful tool for global IADL assessment. The Lawton and Brody IADL scale delivered mixed results. Jefferson *et al*. detected no significant differences between MCI and healthy controls [43], whereas Pedrosa *et al*. found large effects [45] and Mariani *et al*. discovered small effects [44]. The same holds true for the Bayer-ADL. Large effects were seen in the two studies by Perneczky *et al*. [46,47], but only small effects were reported in the studies by Reppermund *et al*. [28,29].

#### Self-report rating instruments

Using the Seoul-IADL in a self-rating version, Kim *et al*. [36] found patients with MCI to be significantly impaired in using a telephone, keeping appointments, talking about recent events and using household appliances, thus replicating the findings of Ahn *et al*. with the Seoul-IADL in an informant-rating version [41]. In addition, Kim *et al*. also reported worse performance of the MCI group for transportation and finances. Peres *et al*. [27] investigated restriction to four IADL items from the Lawton and Brody IADL scale in a self-rating version: telephone use, mode of transport, medication responsibility and handling finances. Patients with MCI were more often restricted in IADL (34.3%) than controls (5.4%) and were less restricted than patients with dementia (91.1%). Interestingly, within a 2-year period, IADL-restricted patients with MCI converted to dementia more frequently than IADL-nonrestricted patients with MCI (30.7% versus 7.8%).

### Global instrumental activities of daily living and mild cognitive impairment subtypes

When we analyzed MCI subtypes, differences between MCI subtypes and normal controls were reported for all applied measures except of the Lawton and Brody IADL scale. Looking at effect sizes, the IADL deficits tended to be more pronounced in multiple-domains MCI than in single-domain MCI and also in amnestic MCI than in nonamnestic MCI.

#### Informant-report rating instruments

Focusing on MCI subtypes, Tam *et al*. [48] found that the multiple-domains MCI subgroup had an intermediate IADL performance level between those of normal controls and patients with mild dementia on the Disability Assessment for Dementia (DAD) scale. Using the DAD, IADL performance, as well as subjects’ performance regarding initiation or planning and organizing of the IADL subtasks, can be evaluated. The amnestic MCI group had significantly better IADL scores than the multiple-domains MCI group, and their scores were similar to those of the cognitively normal controls. The IADL subscales most frequently impaired in the multiple-domains MCI group were those connected to planning and organizing IADL tasks; initiation of tasks was unaffected.

Aretouli *et al*. [23] found significant differences between healthy controls and patients with MCI for 12 of 15 items on the Activities of Daily Living-Prevention Instrument. Major difficulties were reported for keeping appointments, using the telephone, remembering current events and finding things at home, and minor difficulties were reported for driving and using transportation, managing finances, organizing and completing activities, and taking medication. An analysis of the MCI subtypes revealed that all four subgroups showed deficits compared with normal controls. However, patients with multiple-domains MCI were not significantly different from those with single-domain MCI, and the amnestic groups did not differ significantly from the nonamnestic groups.

Using the DAD, Yeh *et al*. [33] reported more IADL deficits for both single-domain amnestic MCI und multiple-domains amnestic MCI than for healthy controls. Both MCI groups had better DAD scores than the mild AD group. When they looked at the DAD scores in detail, though, multiple-domains amnestic patients with MCI had deficits on a larger number of items than single-domain amnestic patients with MCI. Applying the DAD-6 (a shortened version of the DAD), de Rotrou *et al*. [40] reported similar findings. Using the FAQ, Teng *et al*. [31] reported better results for normal controls than for patients with MCI. In analyzing the subgroups, they found better results for normal controls than for the amnestic MCI group on all investigated IADL items and better scores than the nonamnestic group on managing bills, preparing taxes, keeping up with current events, attending to media, remembering dates and traveling outside the neighborhood. Luck *et al*. [58] investigated performance on nine IADL items and detected worse performance of patients with MCI compared with healthy controls. Analyses of MCI subtypes revealed that this effect was stronger for amnestic MCI subtypes.

#### Self-report rating instruments

Investigating MCI subtypes and normal controls, Wadley *et al*. [61] found all MCI subgroups reported significantly greater IADL difficulty and worse everyday functioning scores than normal controls at baseline. Over a 3-year period, all MCI groups also showed a significantly steeper decline on the everyday-functioning composite score and IADL performance compared with the cognitively normal group.

#### One study comparing all three assessment modalities

In a study by Burton *et al*. [37], three different IADL measures were used that revealed differences between MCI subtypes and healthy controls on the Scales of Independent Behavior–Revised (on both the self- and informant-report version) and the performance-based Everyday Problems Test. No differences between groups emerged with the use of Lawton and Brody’s IADL scale with either the self-report or the informant-report version.

### Specific instrumental activities of daily living domains

#### Financial capacity performance-based instruments

Financial capacity is the best-studied IADL subdomain. The Financial Capacity Instrument (FCI) has been used in three studies [26,30,32]. The FCI assesses financial capacity in seven domains, including monetary skills, financial concepts and bank statement management. All three studies revealed that the overall financial capacity (total score) of patients with MCI was worse than that of healthy controls. The activity “bank statement management” was consistently affected across studies. Griffith *et al*. [26] additionally found group differences regarding bill payment and financial concepts. Moreover, Triebel *et al*. [32] reported longitudinal data showing that, at baseline, MCI participants were significantly worse than normal controls on all financial domains and on total scores. Furthermore, the MCI group had been divided into converters and nonconverters to dementia. At baseline, the MCI nonconverter group performed better than the converter group in the domains of financial conceptual knowledge, cash transactions, bank statement management, bill payment and both total scores. No differences were observed for the domains of basic monetary skills, checkbook management, financial judgment and investment decision-making. Over a 1-year period, declines in the domain checkbook management and the total score were observed for the converters, but not for the nonconverters or controls [32].

#### Management of everyday technology

##### Performance-based instruments

In 2010, Malinowsky *et al*. [53] used a standardized observation-based tool (Management of Everyday Technology Assessment) to evaluate ability to manage everyday technology (ET; for example, electronic household appliances, remote controls, cell phones) in patients with mild AD or MCI and controls. They found significant differences between all three groups. Patients with MCI performed worse in using technology than healthy controls did, but better than patients with dementia. In a more recent analysis of the same sample by the same work group [38], significant differences were observed only between healthy controls and patients with dementia when intrapersonal and environmental features were controlled for. They reasoned that what influences a person’s ability to use ET—besides cognitive level or diagnosis—is within-person variability in intrapersonal characteristics and environmental influence (that is, the design of the ET and the context in which it is used).

##### Informant-rating instruments

Muñoz-Neira *et al*. [54] added a technology subscale to a Spanish ADL questionnaire. They found significant group differences between healthy controls, patients with MCI and patients with dementia for the total score. Patients with AD had worse scores than patients with MCI and healthy controls on all seven subscales. Comparing patients with MCI and healthy controls, only the recreation and travel subscales differed significantly; no difference was observed for the technology subscale.

##### Self-report rating instruments

Applying the Everyday Technology Use Questionnaire, Rosenberg *et al*. [56] investigated the perceived difficulty in use of everyday technologies in samples with AD, MCI and controls. They found significant differences between groups, as well as in the amount of technologies that were considered relevant in each group. Using the same instrument, Nygård *et al*. [55] could replicate the above-mentioned findings. Furthermore, they found a moderately strong association between engagement in everyday life activities and perceived difficulty in ET use in these three samples.

#### Driving capacity

##### Performance-based instruments

Wadley *et al*. [51] investigated driving ability, which revealed that patients with MCI were significantly more likely than participants who were cognitively normal to be given “less than optimal” ratings for left-hand turns, lane control and the global driving rating. Furthermore, they tended to receive more “less than optimal” ratings on gap judgment and maintaining proper speed. No differences were found for right-hand turns or steering steadiness. The authors noted, however, that the magnitude of difference between MCI participants’ driving performance and that of controls was small, and that, as a group, MCI drivers were not sufficiently impaired to have their driving ability rated as unsafe or unsatisfactory.

##### Self-report rating instruments

O’Connor *et al*. [59] investigated 5-year trajectories of mobility indicators, including driving frequency and perceived driving difficulty. The study revealed that driving frequency had a steeper decline in the MCI group compared with healthy controls. Furthermore, driving in both normal and demanding situations was perceived as more difficult by patients with MCI than controls.

#### Shopping capacity performance-based instruments

Werner *et al*. [52] directly assessed the IADL domain of shopping by means of a virtual reality supermarket scenario (the Virtual Action Planning Supermarket). They found that patients with MCI covered a significantly higher mean distance, had longer pauses and accordingly took longer to complete their shopping than normal controls. However, the number of purchases, correct or wrong actions, stops and mean time to pay did not differ between groups.

## Discussion

This review impressively illustrates that deficits in IADL are consistently present in MCI. Of the 37 included studies, 35 revealed deficits in global IADL or in specific IADL subdomains such as finances, shopping, keeping appointments, driving or ET use. Furthermore, compared with healthy controls, patients with MCI needed longer to complete tasks and tended to be less accurate. Effect sizes were predominantly moderate to large. In analyzing the MCI subtypes, we observed that the IADL deficits tended to be more pronounced in multiple-domains MCI than in single-domain MCI and in amnestic MCI than in nonamnestic MCI, respectively.

In general, patients with MCI had intermediate functional performance between healthy controls and patients with mild AD, particularly in more complex tasks with high cognitive demand. Financial capacity, particularly, was affected in a vast majority of studies. On the general IADL questionnaires, telephone use, responsibility for medication and keeping appointments were the domains most often affected. Nevertheless, there were studies that revealed no deficits in these domains [37,42]. Even when comparing studies in which researchers used the same instrument, such as the Seoul-IADL [36,41], only three matching domains emerged: telephone use, keeping appointments and using household appliances. Similar inconsistencies were observed for Lawton and Brody’s IADL Scale [5]. In two studies in which this instrument was used, investigators did not find any differences between patients with MCI and persons who were cognitively normal [37,43], supporting the argument that this scale is not sensitive enough to detect subtle deficits in MCI. However, researchers in two other studies [44,45] used the same scale and identified impairments in patients with MCI regarding the domains of shopping, medication and finances. One possible explanation for these inconsistencies is the very heterogeneous operationalization of the MCI criteria. Some studies relied solely on a clinical decision, and others used cutoff scores to determine the magnitude of cognitive impairment, but even the cutoff scores varied between 1 SD and 1.5 SD below age- and education-adjusted norms. Furthermore, the mean MMSE scores of MCI subjects ranged from 23.1 [36] to 28.7 points [37], and mean MMSE scores of normal controls ranged from 26.5 [36] to 29.4 points [30]. The problem with studies including patients with MCI with very low MMSE scores is that IADL deficits may be due to already present, but not yet diagnosed, dementia. In a long-term study of patients with mild AD (MMSE score range, 20 to 26), 45% to 65% could not perform usual IADL tasks at baseline, and 70% to 85% of the remaining patients needed assistance with IADL after 3 years [81]. For future research, it would be helpful to conduct (sub)analyses with patients with MCI who have a MMSE score of 27 points or higher to ensure that they have not already converted to dementia. Another possibility is to use cutoff scores of 1 SD, instead of 1.5 SD, below age- and education-adjusted norms in neuropsychological tests [82]. Moreover, it should be taken into consideration that the MMSE is a rather insensitive measure for cognitive functioning, as it is not adjusted for age and education. In general, the use of MMSE cutoff scores to define MCI should be scrutinized.

In reviewing the selected articles, we found that the variety of assessment instruments applied to assess IADL in MCI was impressive; 31 different instruments were identified (see Table 1), which complicates comparisons among studies. Another problem is that few of these instruments were constructed and validated for IADL assessment in patients with MCI. The majority of the instruments used were originally designed for studies with patients with dementia, and thus the items are not calibrated to detect subtle differences from normal. Moreover, data on psychometric properties are mainly insufficient; for an overview of IADL scales in dementia where the need for validation studies is explicated, see the article by Sikkes *et al*. [83]. Measures specifically designed for MCI populations are required. This may be exemplified by the failure of the ADCS-ADL scale to reveal differences between patients with MCI and healthy controls [25], whereas the ADCS-MCI-ADL scales definitely detected differences [46,47]. The problem could be solved by constructing more sensitive item scoring for MCI-specific scales and/or by investigating in detail only those domains that have been shown to be impaired consistently in MCI, such as financial capacity. When the domain of financial capacity was thoroughly analyzed by an interview or a performance-based assessment procedure, differences between patients with MCI and control participants with cognitive impairment were persistently observed [26,32,39] and invariably revealed large effect sizes.

Furthermore, the majority of assessment instruments do not investigate computer skills or the handling of “new” technology in general. The instruments targeting ET use are examples of scales that focus on a particular domain that proved to be sensitive to subtle impairment, and significant differences were detected through both self-reports and observations [53-56].

Performance-based assessment methods seem to be a promising tool, especially for patients without proxies to provide information about the patient’s IADL. Moreover, performance-based methods would overcome another methodological issue related to self- and/or informant-report measures. In three reviewed studies [15,25,44], healthy controls rated their IADL capacity themselves, whereas MCI subjects were rated by their proxies. This inconsistency could lead to biased results, as rating procedures differed. All assessment methods have their limitations. When using self-report, patients tend to over- or underestimate their abilities and may not have full insight into the impairments caused by the disease. Informant-based methods rely on the informant’s knowledge about the patient, which might be affected by the amount of care provided. In addition, family members tend to misjudge the patient’s capacity. Performance-based instruments also have limitations, such as a higher degree of training needed by assessors, a more time-consuming evaluation and an unfamiliar environment that might bias the functional performance [84].

Furthermore, this review revealed some main problems of MCI definition. The operationalization of MCI is not clearly specified, which leads researchers to define cutoff points and choose assessment instruments of their own. The new criteria for prodromal AD/MCI due to AD may overcome this problem by including biomarkers for the diagnosis of the condition [85]. Nevertheless, the differentiation between MCI and dementia, as described in the new National Institute on Aging and Alzheimer’s Association criteria, rests on the determination of whether there is significant interference in the ability to function at work or in usual daily activities [86]. Therefore, the identification of IADL deficits in MCI as an early phase of AD is absolutely essential for clinical practice. Regarding the effect sizes, the differences between MCI subjects and healthy controls are not only statistically significant but also clinically relevant and can be considered quite robust. Defining a threshold of functional impairment, however, remains a difficult task. MCI is primarily a neuropsychologically defined construct. To give recommendations on exact thresholds, IADL measures which are specifically designed for and/or validated in MCI populations are needed first. If this is achieved, future criteria for MCI could postulate mild deficits in IADL functioning (that is, more than 1.5 standard deviations below healthy controls) in at least one of the following domains: financial abilities, keeping appointments, task completion time, task accuracy or remembering recent events.

It appears evident on the basis of this review that patients with MCI with IADL deficits are more likely to convert to dementia than are patients with MCI without IADL restrictions [27,32]. In fact, the presence of acquired IADL disability not due to a concomitant physical condition seems to be in itself a valid marker of prodromal AD. Studies assessing structural brain functioning and IADL impairment in MCI simultaneously [87] can help to identify relevant biomarkers of IADL deficits and at-risk individuals. Failure to detect an individual’s functional impairments might preclude training of these activities by occupational therapy or lead to neglecting needs and providing an inadequate amount of care from community-based services. Deterioration in IADL abilities, rather than cognition impairments, predicted a greater need of home help services in AD [88].

## Conclusions

Although there was no uniform agreement about which IADL domains are typically—that is, characteristically and/or specifically—impaired in MCI and which types of instruments may detect those best, a clear tendency nevertheless emerged, with activities requiring higher cognitive processes being consistently affected. Also, the use of performance-based measures and technology-related items seems to be promising.

Future research should concentrate on both the thorough validation of established instruments and the development of new ones. As new instruments for IADL functioning in MCI are being developed, researchers should include items measuring the domains of financial capacities, keeping appointments, task completion time and task accuracy. Moreover, studies comparing the three assessment modalities—that is, self-report, informant-report rating and performance-based—in the same sample are needed. In the long run, this could lead to a more precise definition of functional impairment in MCI in terms of quantifiable cutoff scores.

## Abbreviations

AD: Alzheimer’s disease
ADCS-ADL: Alzheimer’s Disease Cooperative Study/Activities of Daily Living Inventory
ADCS-MCI-ADL-18: 18-item Alzheimer’s Disease Cooperative Study/Activities of Daily Living Inventory adapted for patients with mild cognitive impairment
ADCS-MCI-ADL-24: 24-item Alzheimer’s Disease Cooperative Study/Activities of Daily Living Inventory adapted for patients with mild cognitive impairment
ADL: Activities of daily living
ADL-PI: Activities of Daily Living-Prevention Instrument
am: Amnestic multiple domain
aMCI: Amnestic mild cognitive impairment
as: Amnestic single domain
BADL: Basic activities of daily living
Bayer-ADL: Bayer Activities of Daily Living Scale
CDR: Clinical dementia rating
DAD: Disability Assessment for Dementia
DAD-6: 6-item Disability Assessment for Dementia
DAFS: Direct Assessment of Functional Status
DHQ: Driving Habits Questionnaire
DOT: Day-Out Task
EPT: Everyday Problems Test
ETUQ: Everyday Technology Use Questionnaire
FAQ: Functional Activities Questionnaire
FC-ADL: Functional Capacities for Activities of Daily Living
FCI: Financial Capacity Instrument
FC-IADL: Functional Capacities for Instrumental Activities of Daily Living
IADL: Instrumental activities of daily living
4-IADL: 4-item Instrumental Activities of Daily Living scale items chosen from Lawton and Brody
9-IADL: 9-item Instrumental Activities of Daily Living scale
ICC: Intraclass correlation coefficient
IQCODE: Informant Questionnaire on Cognitive Decline in the Elderly
KI-IADL: Knowledgeable Informant report about Instrumental Activities of Daily Living
L&B IADL: Lawton and Brody’s Instrumental Activities of Daily Living
MCI: Mild cognitive impairment
md: Multiple domain
META: Management of Everyday Technology Assessment
MMSE: Mini Mental State Examination
nam: Nonamnestic multiple domain
naMCI: Nonamnestic mild cognitive impairment
nas: Nonamnestic single domain
NAT: Naturalistic action task
NC: Normal control
NIA-AA: National Institute on Aging and Alzheimer’s Association
ns: nonsignificant
ROIL: Record of Independent Living
sd: Single domain
SD: Standard deviation
S-IADL: Seoul-Instrumental Activities of Daily Living
SIB-R: Scales of Independent Behavior–Revised
SR-IADL: Self-report Instrumental Activities of Daily Living
TADL-Q: Technology–Activities of Daily Living Questionnaire
TFLS: Texas Functional Living Scale
TIADL: Timed Instrumental Activities of Daily Living
UAB-DA: University of Alabama at Birmingham Driving Assessment
UCSD-UPSA: University of California San Diego Performance-Based Skills Assessment
VAPS: Virtual Action Planning Supermarket
